# An Optimized Synthesis, Molecular Structure and Characterization of Benzylic Derivatives of 1,2,4-Triazin-3,5(2*H*,4*H*)-dione

**DOI:** 10.3390/molecules22111924

**Published:** 2017-11-08

**Authors:** Long-Chih Hwang, Shiun-Yau Yang, Chung-Lin Chuang, Gene-Hsiang Lee

**Affiliations:** 1 Department of Medicinal and Applied Chemistry, College of Life Sciences, Kaohsiung Medical University, Kaohsiung 807, Taiwan; hangtenyang@gmail.com (S.-Y.Y.); jojodick1215@gmail.com (C.-L.C.); 2 Department of Medical Research, Kaohsiung Medical University Hospital, Kaohsiung 807, Taiwan; 3 Instrumentation Center, College of Science, National Taiwan University, Taipei 106, Taiwan; ghlee@ntu.edu.tw

**Keywords:** 1,2,4-triazine, 1,2,4-triazin-3,5(2*H*,4*H*)-dione, 6-azauracil, X-ray crystal structure, short contacts, synthesis

## Abstract

4-Benzyl-1,2,4-triazin-3,5(2*H*,4*H*)-dione (3-benzyl-6-azauracil, **2**), and 2,4-dibenzyl-1,2,4-triazin-3,5(2*H*,4*H*)-dione (1,3-dibenzyl-6-azauracil, **3**) were synthesized by the reaction of 1,2,4-triazin-3,5(2*H*,4*H*)-dione (6-azauracil, **1**) with benzyl bromide and potassium carbonate in dry acetone via the 18-crown-6-ether catalysis. In these reaction methods, we developed more convenient and efficient methodologies to afford compounds **2** and **3** in good yields. These compounds were characterized by ^1^H- and ^13^C-NMR, MS spectrum, IR spectroscopy and elemental analysis. The structure of **2** was verified by 2D-NMR measurements, including gHSQC and gHMBC measurements. A single-crystal X-ray diffraction experiment indicated that compound **3**, with the molecular formula C_17_H_15_N_3_O_2_, crystallized from a CH_3_OH/CH_2_Cl_2_ diffusion solvent system in a monoclinic space group *P*2_1_/*c* with *a* = 13.7844(13), *b* = 8.5691(8), *c* = 13.0527(12) Å, *β* = 105.961(2)°, V = 1482.3(2) Å^3^, Z = 4, resulting in a density D_calc_ of 1.314 g/cm^3^. The crystal structure of compound **3** is tightly stabilized by contact with five other molecules from the six short contacts formed by intermolecular C−O···H−C*ar*, C−H···C*ar*, and weakly π···π stacking interactions. The dihedral angle 31.90° is formed by the mean planes of the benzene rings of the N-2 and N-4 benzyl groups.

## 1. Introduction

Many aza/deaza analogues of purine have been found to display interesting biological activities. The 1,2,4-triazine ring system had been suggested for study because of various interesting biological activities [1]. Until now, the 1,2,4-triazine and/or fused 1,2,4-triazine moiety have been two of the most popular heterocycles in heterocyclic chemistry. They have been reported to possess a broad spectrum of biological properties and are used in medicine. In the last decade numerous compounds with the 1,2,4-triazine moiety have been synthesized, along with numerous biological tests, thus revealing their varied bioactivity, such as benzodiazepine receptor inhibition [2], antimicrobial [3,4], anxiolytic [5,6], neuroprotective [7], antifungals [8], anti-inflammatory [6,9], antiparasitic [10], antiparkinson [11], and anticancer [12,13,14,15] activities.

The starting material of the title compound 1,2,4-triazin-3,5(2*H*,4*H*)-dione (6-azauracil; **1**), an azapyrimidine analogue of uracil, has been known for over 60 years [16] to inhibit the growth of various species of microorganisms. 6-Azauridine, the ribonucleoside of 6-azauracil, has been proved to display a range of biological effects which include antiviral [17,18], antitumor [19,20], and antifungal [21] activities. The major effect has been ascribed to the in vivo formation of 6-azauridine-5′-phosphate (6-azaUMP), a competitive inhibitor of orotidine-5′-phosphate (OMP) decarboxylase. OMP decarboxylase catalyzes the decarboxylation of orotidine-5′-phosphate to uridine-5′-phosphate, an essential step in the de novo biosynthesis of pyrimidine nucleoside [22,23]. Meanwhile, 6-azaUMP is also a strong inhibitor of inosine-5′-monophosphate dehydrogenase (IMP dehydrogenase), which catalyzes the rate-limiting reaction of de novo GTP biosynthesis [24].

6-Azauracil and its derivatives form an important class of heteroaromatic compounds with various interesting biochemical properties [1]. Among these derivatives the 2-benzyl-1,2,4-triazin-3,5(2*H*,4*H*)-dione has showed anticoccidial activity [25] and property of aldose reductase inhibitor [26]. Banavara [25] reported the product yields were in 10–40% range in synthesis 2-substituted, 4-substituted, or symmetrically 2,4-disubstituted 1,2,4-triazin-3,5(2*H*,4*H*)-dione compounds, and the yield of synthesis of unsymmetrically 2,4-disubstituted compounds were showed in 20–60%. In our past studies on acyclonucleosides synthesis the site of glycosylation was established at the N-2 position of the 2-substituted products of 1,2,4-triazin-3,5(2*H*,4*H*)-dione, based on the comparison of the UV spectra and identified by ^1^H-^13^C heteronuclear correlation (HETCOR) NMR experiments [27,28,29]. Meanwhile, our studies increased the 2-substituted products yield more than 70% by modifying the silyl-Hilbert-Johnson (SHJ or Vorbrüggen) reaction [30]. In this work, we investigated the preparation methods of 4-substituted and symmetrically 2,4-disubstituted of 1,2,4-triazin-3,5(2*H*,4*H*)-dione. Furthermore, we report advanced information about the structure of 4-substituted molecule **2** by spectral analysis, mainly 2D-NMR. In addition, the full characterization by X-ray structural analysis of the molecule **3** is also reported. These improved synthesis methods and structure character will facilitate our preparation and identification of 2-, 4-substituted, and symmetrically, unsymmetrically 2,4-disubstituted compounds of 1,2,4-triazin-3,5(2*H*,4*H*)-dione as potential bioactive molecules in our ongoing program.

## 2. Results and Discussion

### 2.1. Chemistry

**Scheme 1:**
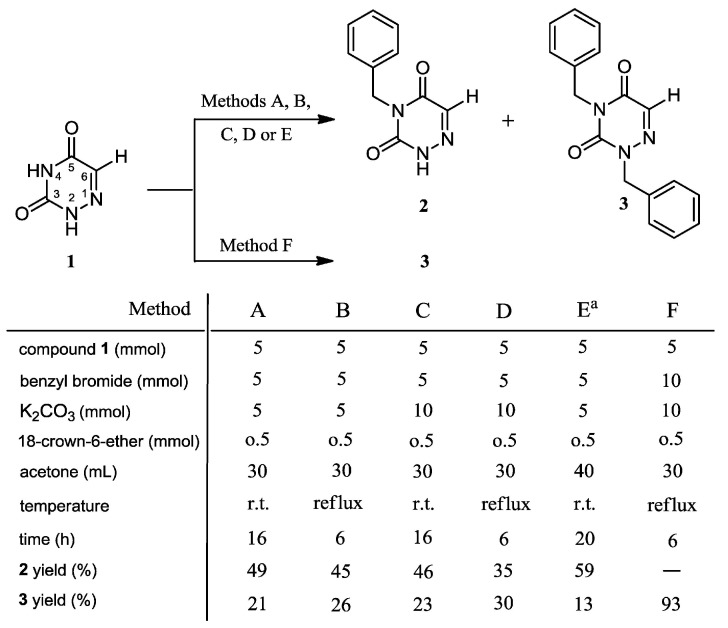
The IUPAC numbering system of compound 1,2,4-triazin-3,5(2*H*,4*H*)-dione (6-azauracil; **1**), synthesis of the 4-benzyl-1,2,4-triazin-3,5(2*H*,4*H*)-dione (**2**) and 2,4-dibenzyl-1,2,4-triazin-3,5(2*H*,4*H*)-dione (**3**) to afford different yield ratios by different methods. ^a^ Benzyl bromide in dry acetone (15 mL), which was added to the flask five times, each time with 3 mL drop by drop in 20 min for 2 h, stirred at room temperature for a total of 20 h.

The preparation of 4-benzyl-1,2,4-triazin-3,5(2*H*,4*H*)-dione (**2**) and 2,4-dibenzyl-1,2,4-triazin-3,5(2*H*,4*H*)-dione (**3**) are illustrated in Scheme 1. The starting material 1,2,4-triazin-3,5(2*H*,4*H*)-dione (6-azauracil; **1**) was prepared according to the method described by Novacek [31]. Scheme 1 presents the IUPAC numbering system of compound **1** reacted with potassium carbonate and benzyl bromide in dry acetone at room temperature for 16 h, under 18-crown-6-ether catalysis, to afford a 2.3:1 ratio of major product **2** (49%; *R*_f_ = 0.42, chloroform:methanol = 18:1) and minor product **3** (21%; *R*_f_ = 0.63) (Method A). Keeping the same amount of each reagent and solvent volume, we refluxed the mixture for 6 h, affording compounds **2** (45%) and **3** (26%) (Method B). The same condition as Method A, except the anhydrous potassium carbonate with two equivalents, would afford compounds **2** (46%) and **3** (23%) (Method C). Moreover, the same condition as Method B, except the anhydrous potassium carbonate with two equivalents, gave compounds **2** (35%) and **3** (30%) (Method D). With a modified Method A (i.e., under the same amount of each reagent, using a pressure-equalizing dropping funnel with the benzyl bromide in dry acetone, which was introduced drop by drop into a round-bottomed flask, the total solvent volume increase 33%, reacted at room temperature for 20 h), we obtained compounds **2** (59%) and **3** (13%) (Method E). This reaction method increased the yield of **2** significantly and decreased the yield of **3**. In method E, the lower concentration of benzyl bromide added to react with compound **1** make the yield of the N-4 benzylated much higher than the N-2,N-4 dibenzylated, which implies N-4 nucleophile have more opportunity to react with benzyl bromide. In other words, the evidence showed the dissociation rate of N-4-H is quicker than N-2-H of compound **1** under basic conditions. In another modified reaction of Method A, we increased potassium carbonate and benzyl bromide to double amount and refluxed the mixture for 6 h to give the only product 2,4-disubstituted compound **3** in good yield (93%) (Method F). All reaction times of the above methods were monitored by TLC. The improvement of this synthetic method was derived from our previous research [32].

The structures of **2** and **3** were completely assigned by ^1^H- and ^13^C-NMR, MS and IR spectra and EA. In NMR spectroscopy the presence of a benzyl group and eight carbon signals were observed in compound **2**. The thirteen carbon signals showed two benzyl groups of compound **3**. A detailed 2D-NMR study, including gHSQC and gHMBC measurements was necessary to confirm the benzylation sites of **2**. The long-range gHMBC ^1^H-^13^C correlations showed the attachment of the benzyl group on N-4 for structure **2**, since the methylene protons (*δ*_H_ 5.07 ppm) of benzyl group showed a ^3^*J* (H,C) coupling to the C-3 (*δ*_C_ 149.35 ppm) and the C-5 (*δ*_C_ 155.86 ppm) carbonyl carbon atom. Meanwhile, the fact that the proton of the H-6 (*δ*_H_ 7.41 ppm) have a ^2^*J* (H,C) coupling to the C-5 carbon atom and certification the carbon chemical shifts of C-5 is higher than the C-3 carbon in the 1,2,4-triazin-3,5(2*H*,4*H*)-dione moiety. The standard range gHSQC ^1^H-^13^C experiments showed the methylene protons (*δ*_H_ 5.07 ppm) of the benzyl group with a ^1^*J* (H,C) coupling to the methylene (*δ*_C_ 43.35 ppm) carbon atom and H-6 proton have a coupling to the C-6 (*δ*_C_ 135.07 ppm) carbon atom.

**Scheme 2:**
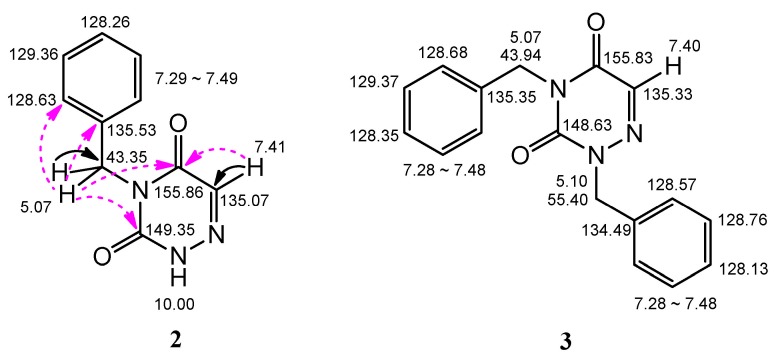
The gHSQC and gHMBC measurements of compound **2** and the chemical shift (*δ*) assignment of ^1^H- and ^13^C-NMR for **2** and **3**.

Scheme 2 presents the complete assignment of ^1^H- and ^13^C-NMR signals for compounds **2** and **3**, together with the corresponding long-range (pink dashed arrow) and short-range (black color) ^1^H-^13^C correlations. The *δ* values of ^1^H- and ^13^C-NMR of 1,2,4-triazin-3,5(2*H*,4*H*)-dione moiety were similar in **2** and **3**. In compound **3**, the *δ* values of the ^1^H-NMR spectra were very similar between the methylene proton on N-2 benzyl and N-4 benzyl group showed *δ* values of 5.10 and 5.07 ppm, respectively. On the other hand, the clearly different *δ* values of the ^13^C-NMR spectra between the methylene carbon on N-2 and N-4 benzyl groups showed *δ* values of 55.40 and 43.94 ppm, respectively. Taken together, the *δ* values of the ^1^H- and ^13^C-NMR spectra display the methylene group on N-2 showed significant downfield shifts than the N-4 benzyl group of molecule **3**.

### 2.2. Analysis of the X-ray Crystallographic Structure

**Figure 1:**
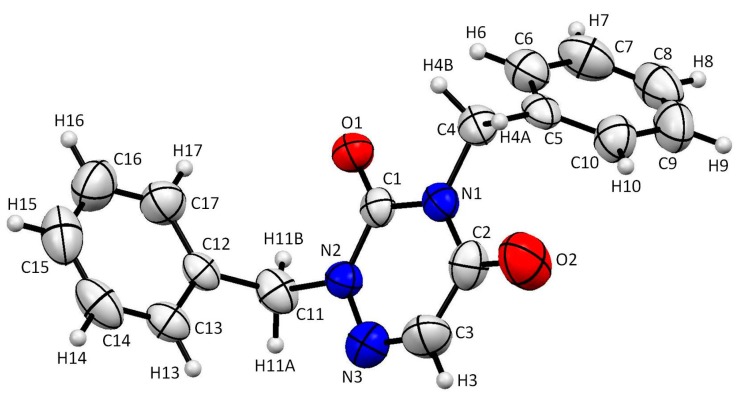
An ORTEP drawing of compound **3** with atom labelling; thermal ellipsoids are drawn at the 50% probability level.

An ORTEP drawing for the molecular **3** is depicted in Figure 1. From this X-ray analysis of the Compound **3**, revealing the 1,2,4-triazine ring is slightly distorted. Obviously, the benzyl groups are located at N2 (i.e., N-2) and N1 (i.e., N-4). The location of benzyl group are compatible with tautomeric proton 2-H located at N-2 and 4-H located at N-4 of the starting material 1,2,4-triazin-3,5(2*H*,4*H*)-dione (**1**). The bond lengths and angles for compound **3** are shown in Table S1 (see Supplemental Material).

In the crystal structure of **3** of the 1,2,4-triazin-3,5(2*H*,4*H*)-dione moiety, the shorter bond 1.273(6) Å of N3–C3 has an appreciable double-bond character and the longest bond 1.441(7) Å is C2–C3. The bond length 1.211(4) Å of C1–O1 and 1.211(5) Å of C2–O2 are shorter than the bond length 1.240 Å of Csp^2^=O in *δ*-lactams [33], due to the strong electron withdraw by these nitrogen atoms in the 1,2,4-triazine ring. Additionally, the sum of these three angles, O2–C2–N1, O2–C2–C3, and N1–C2–C3, as well as the sum of O1–C1–N2, O1–C1–N1, and N2–C1–N1, both reach 360°, thus confirming the sp^2^ hybridization bonding. Meanwhile, the bond angle 123.0(4)° of N1–C1–O1 is wider than N1–C2–O2 and N2–C1–O1. This evidence suggests that the lone electron pair of the two nitrogen atoms N1 and N2 strongly resonates with O2 and O1, respectively. Additionally, the angles 112.6(3)° of N1–C4–C5 and 113.3(3)° of N2–C11–C12 are similar through the N-4 and N-2 benzyl group, respectively. The dihedral angles of 66.52° and 68.53° forms from mean plane of the central ring (defined by atoms N1, N2, N3, C1, C2, and C3) with mean planes of the benzene rings C5–C10 and C12–C17 of the N-4 and N-2 benzyl groups, respectively (Figure S1) (see Supplemental Material). Meanwhile, another dihedral angle 31.90° is formed by mean planes of the benzene rings of the N-2 and N-4 benzyl groups (Figure S1).

### 2.3. Analysis of the Molecular Packing

**Figure 2:**
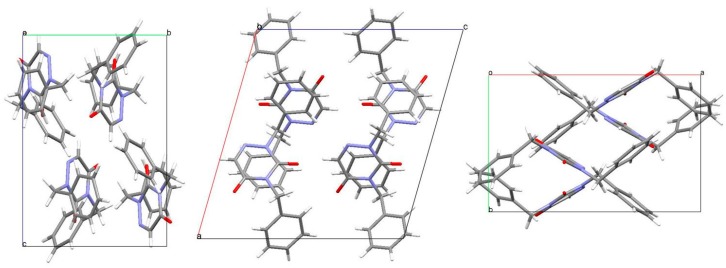
A perspective drawing of the packing arrangement of compound **3**, showing the molecules’ direction along the a, b, and c axis.

**Figure 3:**
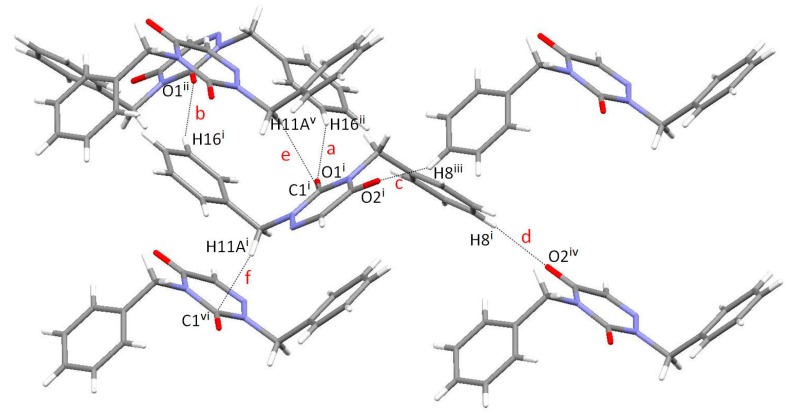
Intermolecular short contacts in compound **3** along the c axis. For the notation and symmetry codes see the text.

The molecular packing of compound **3** is shown in Figure 2. An analysis of the molecular packing in the unit cell reveals that there are neither classic hydrogen bond nor molecule disorder in the crystal structure of **3**. The intermolecular short contacts in the molecular graphic were all obtained using the Mercury program (version 2.3, CCDC, Cambridge, UK). We note that in the unit cell each molecule is linked to five other molecules with six short contacts (Figure 3). These short contacts include weak hydrogen bond interactions (Figure 3 notation (a, b, c, d)) and C-sp^2^···s-sp^3^ interactions (Figure 3 notation (e, f)) which stabilized the crystal structure. The title molecule (*i* = *x*, *y*, *z*) atom C1–O1*^i^* linked with H16–C*ar^ii^* at (*ii* = 1 − *x*, −*y*, 1 − *z*) and C*ar*–H16*^i^* linked with O1–C1*^ii^* with an identical short contacts distance of 2.656(3) Å (Figure 3 notation (a, b)). Meanwhile, the Oxygen atom C2–O2*^i^* linked with H8−C*ar^iii^* at (*iii* = 2 − *x*, −1/2 + *y*, 1/2 − *z*) and C*ar*–H8^i^ linked with O2–C2*^iv^* at (*iv* = 2 − *x*, 1/2 + *y*, 1/2 − *z*) have a same short contacts distance of 2.566(5) Å (Figure 3 notation (c, d)). Another type of short contact involves interactions between layer and layer via O1–C1^i^ linked with H11A–C11*^v^* at (*v* = 1 − *x*, −1/2 + *y*, 1/2 − *z*) and H11A–C11*^i^* linked with C1–O1*^vi^* at (*vi* = 1 − *x*, 1/2 + *y*, 1/2 − *z*) with an identical contact distance of 2.771(4) Å (Figure 3 notation (e, f)). The bonding environment of carbon C1*^i^* is in a π-deficient 1,2,4-triazinone ring and also attaches with oxygen O1*^i^*, resulting more positive charge in the C1*^i^* (C-sp^2^), which forms a short contact with H11A−C11*^v^* (s-sp^3^). Another possible interpretation is that the C11–H11A···C1 involves an sp^3^ C atom and likely exhibits the observed motif because of close packing principles rather than some cohesive interaction.

**Figure 4:**
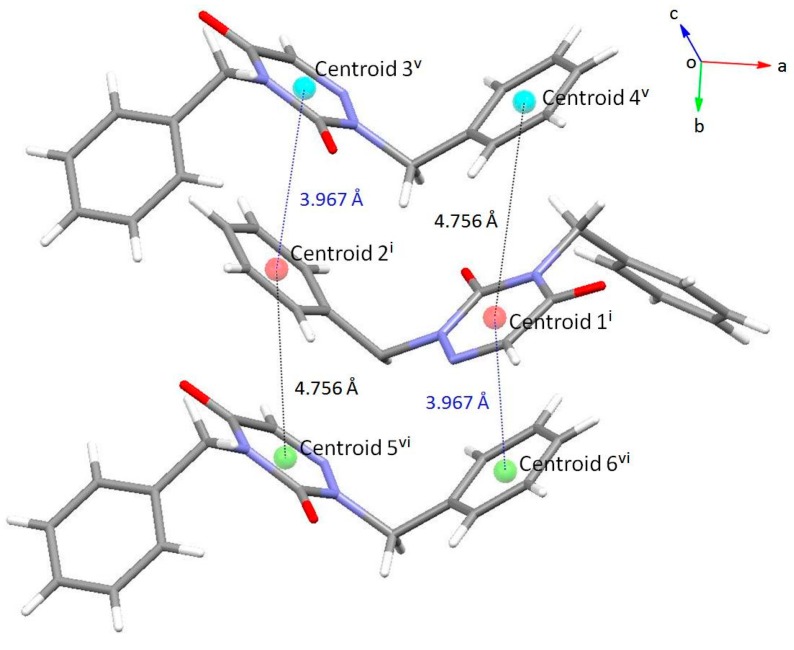
A view of π···π stacking interactions contact running parallel to the [010] direction in compound **3** along the c axis. For the symmetry codes see the text.

In addition to the above three types of short contacts, it is worth noting that the crystal structure is further stabilized by weakly π···π stacking interactions (see Figure 4). The centroid 1*^i^* (defined by atoms N1N2N3C1C2C3 of the molecule **3**, *i* = *x*, *y*, *z*), contacts with the centroid 4*^v^* (defined by atoms C12–C17 of the upper layer molecule, *v* = 1 − *x*, −1/2 + *y*, 1/2 − *z*) with a π···π stacking interactions distance 4.756 Å. Meanwhile, the centroid 2*^i^* (atoms C12–C17) contacts with centroid 5*^vi^* (atoms N1N2N3C1C2C3, *vi* = 1 − *x*, 1/2 + *y*, 1/2 − z) of the lower layer molecule with the same contact distance 4.756 Å. Another π···π stacking interactions with contact distance of 3.967 Å are centroid 1*^i^* contacts with centroid 6*^vi^* (atoms C12–C17) and centroid 2*^i^* contacts with centroid 3*^v^* (atoms N1N2N3C1C2C3).

## 3. Materials and Methods

### 3.1. General Techniques

Melting points were measured on a Yanaco MP-500D micromelting point apparatus (Yanaco Technical Science Co., Ltd., Kyoto, Japan) and were uncorrected. The Infrared spectra were recorded as KBr discs on a Perkin-Elmer FTIR 1650 instrument (PerkinElmer, Waltham, MA, USA). The ^1^H-NMR and ^13^C-NMR spectra were obtained in CDCl_3_ on a Varian Mercury-plus 400 (400 MHz) spectrometer (Varian Inc., Palo Alto, CA, USA). Chemical shifts are expressed in ppm (*δ*) with tetramethylsilane (TMS) as an internal standard. For the assignments of signals, standard and long-range ^1^H-^13^C heteronuclear chemical shift correlation 2D-NMR experiments (gHSQC and gHMBC) were used. Thin layer chromatography (TLC) analyses were performed on silica gel plates (Merck 60 F_254_, 0.2 mm thickness; Merck KGaA, Darmstadt, Germany), and the components were detected by UV light (254 nm). Mass spectra were obtained on a gas chromatography mass spectrometer (GC-MS) Thermo Finnigan PolarisQ quadrupole ion trap (Thermo electron corporation, Austin, TX, USA). Elemental analyses were performed on a Heraeus CHN-O-Rapid elemental analyzer (Heraeus GmbH, Hanau, Germany). All the solvents used were dried and distilled under argon prior to use.

### 3.2. Syntheses

#### 3.2.1. Syntheses of 4-Benzyl-1,2,4-triazin-3,5(2*H*,4*H*)-dione (**2**) and 2,4-Dibenzyl-1,2,4-triazin-3,5(2*H*,4*H*)-dione (**3**)

Method A: A solution of **1** (0.57 g, 5 mmol) in dry acetone (30 mL) was mixed with anhydrous potassium carbonate (0.69 g, 5 mmol) and a catalytic amount of 18-crown-6-ether (0.13 g, 0.5 mmol). Then benzyl bromide (0.86 g, 5 mmol) was added and the mixture stirred at room temperature for 16 h (monitored by TLC). The solvent was evaporated to afford a crude product which was then applied to a silica gel (230–400 mesh) column. The column was eluted with a mixture of chloroform and methanol (80:1) and the appropriate fractions were combined and evaporated.

The *R*_f_ value of the major adduct was 0.42 (chloroform:methanol = 18:1). The residue thus obtained was recrystallized from dichloromethane and methanol (20:1) which afforded white crystals **2** in 49% yield (0.50 g). m.p. = 118–119 °C; IR (cm^−1^), *δ* max: 3213, 3170, 3092, 2920, 1727 (C=O), 1662 (C=N), 1496, 1442, 1411, 1353, 1210, 1160, 937, 890; ^1^H-NMR (400 MHz, CDCl_3_) *δ* = 5.07 (s, 2H, CH_2_), 7.41 (s, 1H, 6-H), 10.00 (brs, 1H, NH), 7.29–7.35 (m, 3H, Ar-H), 7.46–7.49 (dd, 2H, Ar-H); ^13^C-NMR (400 MHz, CDCl_3_) *δ* = 43.35 (CH_2_), 128.26, 128.63, 129.36, 135.53 (Ar-C), 135.07 (C-6), 149.35 (C-3), 155.86 (C-5); MS, *m*/*z* (%): 203 (M^+^, 53), 147 (100), 132 (9), 104 (28), 91 (32), 77 (17), 65 (17), 51 (15); Anal. Calcd. for C_10_H_9_N_3_O_2_ (203.20): C, 59.11; H, 4.46; N, 20.68; Found: C, 58.89; H, 4.48; N, 20.38.

The *R*_f_ value for the minor product was 0.63 (chloroform:methanol = 18:1). The residue thus obtained was recrystallized from dichloromethane and methanol (30:1) to give **3** as bright white crystals in 21% yield (0.31 g). m.p. = 95–96 °C; IR (cm^−1^), *δ* max: 1714 (C=O), 1668 (C=N), 1583, 1493, 1432, 1421, 1366, 1331, 1244, 1204, 1062, 907, 866; ^1^H-NMR (400 MHz, CDCl_3_) *δ* = 5.07 (s, 2H, CH_2_), 5.10 (s, 2H, CH_2_), 7.40 (s, 1H, 6-H), 7.28–7.40 (m, 3H × 2, Ar-H), 7.46–7.48 (dd, 2H × 2, Ar-H); ^13^C-NMR (400 MHz, CDCl_3_) *δ* = 43.94 (CH_2_), 55.40 (CH_2_), 128.13, 128.35, 128.57, 128.68, 128.76, 129.37, 134.49, 135.35 (Ar-C × 2), 135.33 (C-6), 148.63 (C-3), 155.83 (C-5); MS, *m*/*z* (%): 293 (M^+^, 28), 265 (7), 238 (11), 202 (4), 175 (12), 161 (53), 132 (59), 106 (52), 91 (100), 77 (33), 65 (47), 51 (21); Anal. Calcd. for C_17_H_15_N_3_O_2_ (293.32): C, 69.61; H, 5.15; N, 14.33; Found: C, 69.43; H, 5.17; N, 14.27

Method B: A solution of **1** (0.57 g, 5 mmol) in dry acetone (30 mL) was mixed with anhydrous potassium carbonate (0.69 g, 5 mmol) and a catalytic amount of 18-crown-6-ether (0.13 g, 0.5 mmol). Then benzyl bromide (0.86 g, 5 mmol) was added and the mixture refluxed for 6 h (monitored by TLC). The separation and recrystallization was treated as described in Method A to afford compounds **2** (0.46 g; 45%) and **3** (0.38 g; 26%).

Method C: The same condition as Method A except the anhydrous potassium carbonate with two equivalent (1.38 g, 10 mmol) was applied. The separation and recrystallization was treated as described in Method A to afford compounds **2** (0.47 g; 46%) and **3** (0.34 g; 23%).

Method D: The reacted condition was the same with Method B except the anhydrous potassium carbonate with two equivalent (1.38 g, 10 mmol). The separation and recrystallization was treated as described in Method A to afford Compounds **2** (0.36 g; 35%) and **3** (0.44 g; 30%).

Method E: Equip a dry 150 mL round-bottomed flask at the top arm attached with a pressure-equalizing dropping funnel. Place a solution of **1** (0.57 g, 5 mmol) in dry acetone (25 mL) mix with anhydrous potassium carbonate (0.69 g, 5 mmol) and a catalytic amount of 18-crown-6-ether (0.13 g, 0.5 mmol) in the round-bottomed flask, and fill the dropping funnel with a solution of benzyl bromide (0.86 g, 5 mmol) in dry acetone (15 mL), which was added to the flask five times, each time with 3 mL drop by drop in 20 min for 2 h, and stirred at room temperature for a total of 20 h (monitored by TLC). The separation and recrystallization was treated as described in Method A to afford compounds **2** (0.60 g; 59%) and **3** (0.19 g; 13%).

#### 3.2.2. 2,4-Dibenzyl-1,2,4-triazin-3,5(2*H*,4*H*)-dione (**3**)

Method F: A solution of **1** (0.57 g, 5 mmol) in dry acetone (30 mL) was mixed with anhydrous potassium carbonate (0.69 g, 10 mmol) and a catalytic amount of 18-crown-6-ether (0.13 g, 0.5 mmol). Then benzyl bromide (1.71 g, 10 mmol) was added and the mixture refluxed for 6 h (monitored by TLC). The solvent was evaporated to afford a crude product which was recrystallized from dichloromethane and methanol (25:1) to give bright white crystals (1.36 g, 93%) identical in every respect with compound **3**, which was afforded by Method A above.

### 3.3. X-ray Diffraction

A plate colorless crystal **3** having dimensions of 0.48 × 0.27 × 0.04 mm was obtained by recrystallization from a CH_3_OH/CH_2_Cl_2_ diffusion solvent system and suitable for single-crystal X-ray diffraction measurements. The data were collected on a Bruker Smart APEXCCD diffractometer (Bruker AXS GmbH, Karlsruhe, Germany) with a graphite-monochromated Mo K*α* radiation (*λ* = 0.71073 Å) at 295(2) K. A total of 8796 reflections and 2612 independent reflections (*R*_int_ = 0.0509) were collected within the range of 2.83 < θ < 25.00° by using *ω* scan technique, of which 1758 observed reflections with *I* *>* 2*σ*(*I*) were used in the structural analysis. The crystal and experimental data are given in Table 1.

The crystal structure was solved by direct methods using SHELXS-97 [34] and refined by full-matrix least-squares methods on *F*^2^ using SHELXL-2014/7 [35]. All of the non-hydrogen atoms were refined anisotropically. All hydrogen atom positions were calculated and included in the calculation using the riding atom model. The final cycle of full-matrix least-squares refinement gave *R*_1_ = 0.0851, *wR*_2_ = 0.1922 incorporating the weighting scheme *w* = 1/[*σ*^2^(*F*${}_{o}^{2}$) + (0.0756*P*)^2^ + 1.3197*P*], where *P* = (*F*${}_{o}^{2}$ + 2*F*${}_{c}^{2}$)/3. *S* = 1.085 and (*Δ/σ*)_max_ = < 0.001. The maximum peak on the final difference Fourier map is 0.556 and the minimum peak −0.189 eÅ^−^^3^. The final positional parameters for all non-hydrogen atoms are given in Table S2 (see Supplementary material).

The supplementary crystallographic data for **3** were deposited at the Cambridge Crystallographic Data Centre (CCDC) as CCDC-1565082. These data can be obtained free of charge from the CCDC, 12 Union Road, Cambridge CB2 1EZ, UK (Fax: +44-1223-336033; www: http://www.ccdc.cam.ac.uk/conts/retrieving.html or e-mail: deposit@ccdc.cam.ac.uk).

## 4. Conclusions

In summary, we have reported the simple, convenient, safe, and high yield synthesis methods and molecular structure characterization of N-4 benzylic **2** and N-2,N-4 dibenzylic **3**. The 2D-NMR spectral analysis allowed identification of the benzylated position and complete assignments of various carbons of the molecules N-4 benzylic **2**. Meanwhile, obviously different *δ* values in ^1^H- and ^13^C-NMR spectra between **2** and **3** were found. A single X-ray crystal structure analysis of **3** supplied the crystal structure data and the analysis of the molecular packing showed evidence that the compound **3** was tightly stabilized by contact with five other molecules from six short contacts formed by intermolecular C–O···H–C*ar*, C–H···C*ar*, and weakly π···π stacking interactions. The dihedral angle 31.90° was formed by the mean planes of the benzene rings of the N-2 and N-4 benzyl groups.

## Figures and Tables

**Table 1:** Crystallographic data of fcompound **3**.

Empirical Formula	C_17_H_15_N_3_O_2_
Formula weight	293.32
Temperature	295(2) K
Wavelength	0.71073 Å
Crystal system	Monoclinic
Space group	*P*2_1_/*c*
	*a* = 13.7844(13) Å
Unit cell dimensions	*b* = 8.5691(8) Å, *β* = 105.961(2)°
	*c* = 13.0527(12) Å
Volume	1482.3(2) Å3
Z	4
Density (calculated)	1.314 Mg/m^3^
Absorption coefficient	0.089 mm^−1^
*F*(000)	616
Theta range for data collection	2.831 to 24.999°
Index ranges	‒16 ≤ *h* ≤ 16, −9 ≤ *k* ≤ 10, −15 ≤ *l* ≤ 15
Reflections collected	8796
Independent reflections	2612 (*R*_int_ = 0.0509)
Completeness to *θ* = 24.999°	99.90%
Absorption correction	Semi-empirical from equivalents
Max. and min. transmission	0.756 and 0.659
Refinement method	Full-matrix least-squares on *F*^2^
Data/restraints/parameters	2612/0/199
Goodness-of-fit on *F*^2^	1.085
Final *R* indices (*I* > 2σ(*I*))	*R*_1_ = 0.0851, *wR*_2_ = 0.1922
*R* indices (all data)	*R*_1_ = 0.1281, *wR*_2_ = 0.2139
Largest difference peak and hole	0.566 and −0.189 eÅ^−^^3^

## Supplementary Materials

Supplementary Materials are available online. Table S1: Bond lengths (Å) and bond angles (°) in compound **3**. Table S2: Atomic coordinates (×10^4^) and equivalent isotropic displacement parameters (Å^2^ × 10^3^) for compound **3**. Figure S1: A view of the dihedral angles (°) of the compound **3**. Figure S2: 4-Benzyl-1,2,4-triazin-3,5(2*H*,4*H*)-dione (**2**), (a) ^1^H-NMR spectrum, (b) ^13^C-NMR spectrum, (c) gHSQC spectrum, (d) gHMBC spectrum, (e) MS spectrum, and (f) IR spectrum. Figure S3: 2,4-Dibenzyl-1,2,4-triazin-3,5(2*H*,4*H*)-dione (**3**), (a) ^1^H-NMR spectrum, (b) ^13^C-NMR spectrum, (c) MS spectrum, and (d) IR spectrum.
