# Supplementary material for: An Optimized Synthesis, Molecular Structure and Characterization of Benzylic Derivatives of 1,2,4-Triazin-3,5(2H,4H)-dione
Source: Molecules. 2017 Nov 8;22(11):1924. doi: 10.3390/molecules22111924 (PMC6150235; doi:10.3390/molecules22111924)

An Optimized Synthesis, Molecular Structure and Characterization of Benzylic Derivatives of 1,2,4-triazin-3,5(2*H*,4*H*)-dione

Long-Chih Hwang, Shiun-Yau Yang, Chung-Lin Chuang and Gene-Hsiang Lee

Table S1. Bond lengths (Å) and bond angles (°) in compound **3**.

O(1)-C(1)	1.213(4)
O(2)-C(2)	1.212(5)
N(1)-C(2)	1.376(5)
N(1)-C(1)	1.386(4)
N(1)-C(4)	1.482(5)
N(2)-N(3)	1.368(4)
N(2)-C(1)	1.378(5)
N(2)-C(11)	1.462(5)
N(3)-C(3)	1.274(5)
C(2)-C(3)	1.438(6)
C(3)-H(3)	0.9300
C(4)-C(5)	1.507(5)
C(4)-H(4A)	0.9700
C(4)-H(4B)	0.9700
C(5)-C(10)	1.374(5)
C(5)-C(6)	1.376(6)
C(6)-C(7)	1.390(6)
C(6)-H(6)	0.9300
C(7)-C(8)	1.358(7)
C(7)-H(7)	0.9300
C(8)-C(9)	1.362(7)
C(8)-H(8)	0.9300
C(9)-C(10)	1.378(6)
C(9)-H(9)	0.9300
C(10)-H(10)	0.9300
C(11)-C(12)	1.496(5)
C(11)-H(11A)	0.9700
C(11)-H(11B)	0.9700
C(12)-C(17)	1.380(5)
C(12)-C(13)	1.383(5)
C(13)-C(14)	1.373(6)
C(13)-H(13)	0.9300
C(14)-C(15)	1.375(7)
C(14)-H(14)	0.9300

C(15)-C(16)	1.366(7)
C(15)-H(15)	0.9300
C(16)-C(17)	1.365(7)
C(16)-H(16)	0.9300
C(17)-H(17)	0.9300
C(2)-N(1)-C(1)	122.0(3)
C(2)-N(1)-C(4)	120.0(3)
C(1)-N(1)-C(4)	118.1(3)
N(3)-N(2)-C(1)	125.5(3)
N(3)-N(2)-C(11)	114.9(3)
C(1)-N(2)-C(11)	119.6(3)
C(3)-N(3)-N(2)	115.8(4)
O(1)-C(1)-N(2)	121.5(3)
O(1)-C(1)-N(1)	122.9(3)
N(2)-C(1)-N(1)	115.7(3)
O(2)-C(2)-N(1)	122.1(4)
O(2)-C(2)-C(3)	122.8(4)
N(1)-C(2)-C(3)	115.1(4)
N(3)-C(3)-C(2)	125.8(4)
N(3)-C(3)-H(3)	117.1
C(2)-C(3)-H(3)	117.1
N(1)-C(4)-C(5)	112.6(3)
N(1)-C(4)-H(4A)	109.1
C(5)-C(4)-H(4A)	109.1
N(1)-C(4)-H(4B)	109.1
C(5)-C(4)-H(4B)	109.1
H(4A)-C(4)-H(4B)	107.8
C(10)-C(5)-C(6)	118.2(4)
C(10)-C(5)-C(4)	120.7(4)
C(6)-C(5)-C(4)	121.2(4)
C(5)-C(6)-C(7)	120.6(4)
C(5)-C(6)-H(6)	119.7
C(7)-C(6)-H(6)	119.7
C(8)-C(7)-C(6)	119.8(5)
C(8)-C(7)-H(7)	120.1
C(6)-C(7)-H(7)	120.1
C(7)-C(8)-C(9)	120.5(5)
C(7)-C(8)-H(8)	119.8
C(9)-C(8)-H(8)	119.8
C(8)-C(9)-C(10)	119.7(5)
C(8)-C(9)-H(9)	120.2

C(10)-C(9)-H(9)	120.2
C(5)-C(10)-C(9)	121.3(5)
C(5)-C(10)-H(10)	119.4
C(9)-C(10)-H(10)	119.4
N(2)-C(11)-C(12)	113.3(3)
N(2)-C(11)-H(11A)	108.9
C(12)-C(11)-H(11A)	108.9
N(2)-C(11)-H(11B)	108.9
C(12)-C(11)-H(11B)	108.9
H(11A)-C(11)-H(11B)	107.7
C(17)-C(12)-C(13)	118.2(4)
C(17)-C(12)-C(11)	121.6(4)
C(13)-C(12)-C(11)	120.2(4)
C(14)-C(13)-C(12)	120.6(4)
C(14)-C(13)-H(13)	119.7
C(12)-C(13)-H(13)	119.7
C(13)-C(14)-C(15)	120.3(4)
C(13)-C(14)-H(14)	119.8
C(15)-C(14)-H(14)	119.8
C(16)-C(15)-C(14)	119.4(5)
C(16)-C(15)-H(15)	120.3
C(14)-C(15)-H(15)	120.3
C(17)-C(16)-C(15)	120.5(5)
C(17)-C(16)-H(16)	119.8
C(15)-C(16)-H(16)	119.8
C(16)-C(17)-C(12)	121.1(4)
C(16)-C(17)-H(17)	119.4
C(12)-C(17)-H(17)	119.4
Symmetry transformations used to generate equivalent atoms	

Table S2. Atomic coordinates ($\times 10^4$) and equivalent isotropic displacement parameters ($Å^2 \times 10^3$) for compound **3**.

	x	y	z	U(eq)
O(1)	6432(2)	1395(3)	3812(2)	58(1)
O(2)	7745(3)	-235(5)	1160(3)	101(1)
N(1)	7131(2)	500(4)	2528(2)	46(1)
N(2)	5619(2)	1909(4)	2091(2)	45(1)
N(3)	5519(3)	1830(5)	1020(3)	67(1)

C(1)	6401(3)	1279(4)	2878(3)	43(1)
C(2)	7094(3)	410(5)	1466(3)	58(1)
C(3)	6229(4)	1137(6)	750(4)	74(1)
C(4)	7976(3)	-238(5)	3338(3)	52(1)
C(5)	8851(3)	859(4)	3752(3)	48(1)
C(6)	8959(3)	1682(5)	4682(4)	63(1)
C(7)	9770(4)	2688(6)	5057(4)	79(2)
C(8)	10457(4)	2876(6)	4495(5)	79(2)
C(9)	10369(3)	2061(6)	3577(5)	78(2)
C(10)	9566(3)	1061(5)	3208(4)	64(1)
C(11)	4810(3)	2743(5)	2382(3)	56(1)
C(12)	3990(3)	1694(4)	2526(3)	43(1)
C(13)	3284(3)	1093(5)	1648(3)	54(1)
C(14)	2536(3)	115(5)	1773(4)	70(1)
C(15)	2473(4)	-268(6)	2776(5)	80(2)
C(16)	3165(4)	329(7)	3646(4)	83(2)
C(17)	3915(3)	1290(6)	3525(3)	66(1)
U(eq) is defined as one third of the trace of the orthogonalized U^{ij} tensor.				

Figure S1. A view of the dihedral angles (°) of the compound **3**.

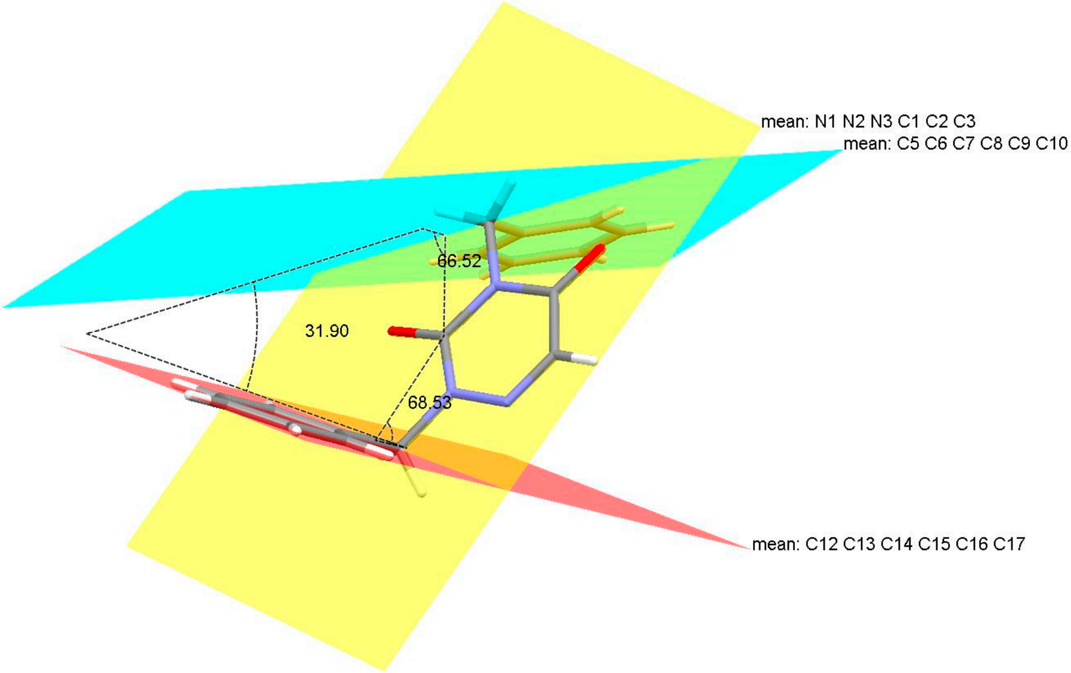

Figure S2: 4-Benzyl-1,2,4-triazin-3,5(2*H*,4*H*)-dione (**2**), (a) ^{1}H NMR spectrum.

Figure S2: 4-benzyl-1,2,4-triazin-3,5(2*H*,4*H*)-dione (**2**), (a) ^{1}H NMR spectrum

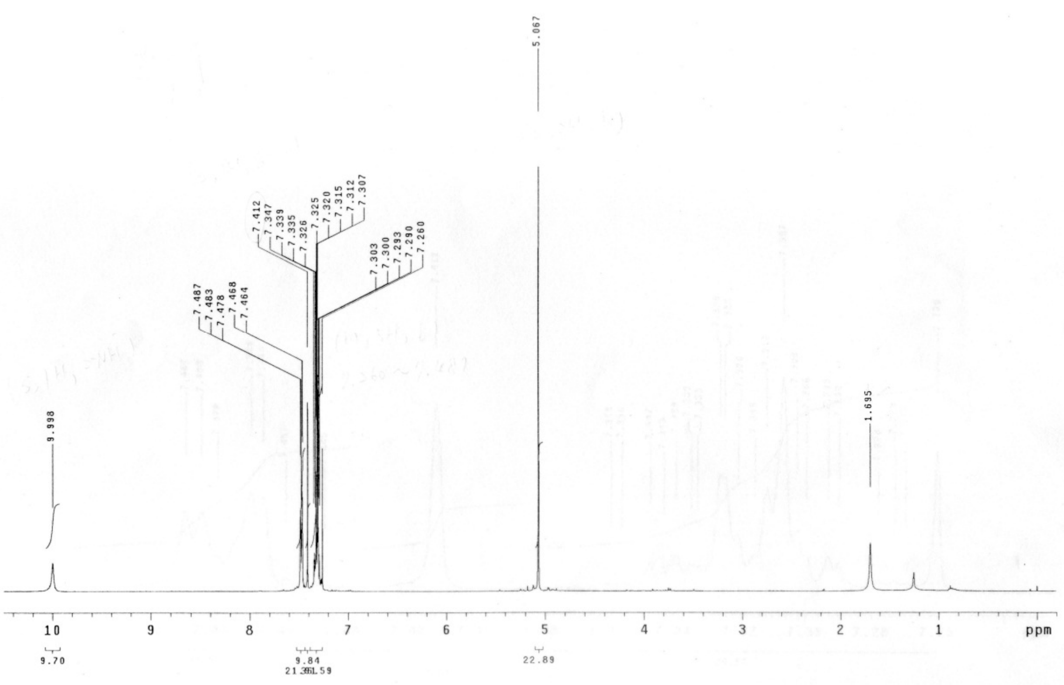

Figure S2: 4-Benzyl-1,2,4-triazin-3,5(2*H*,4*H*)-dione (**2**), (b) ^{13}C NMR spectrum.

Figure S2: 4-benzyl-1,2,4-triazin-3,5(2*H*,4*H*)-dione (**2**), (b) ^{13}C NMR spectrum

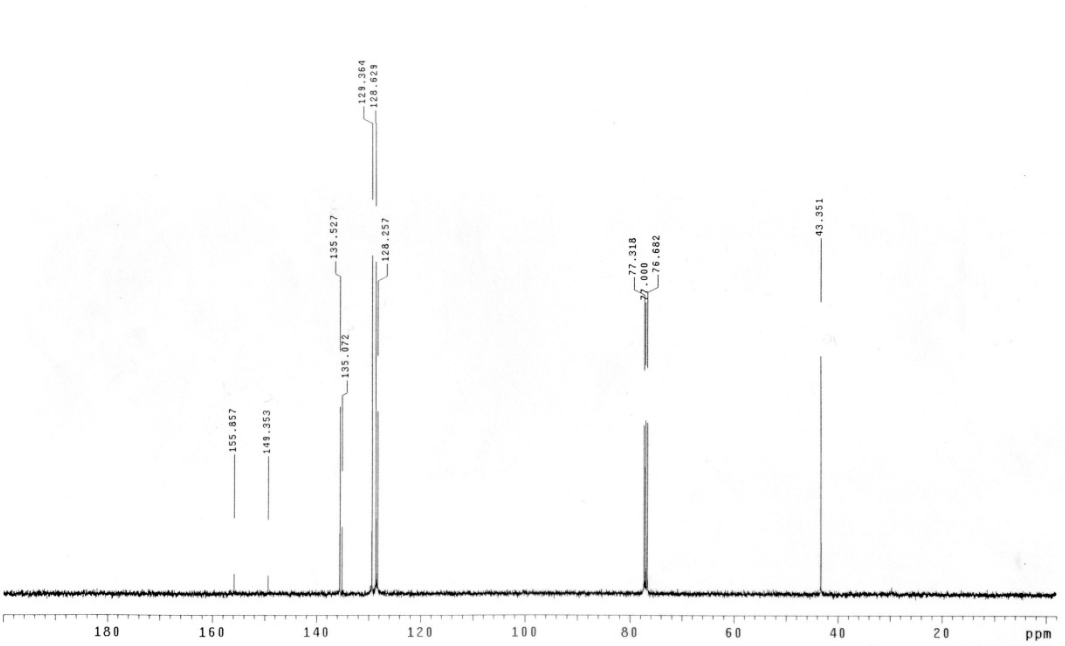

Figure S2: 4-Benzyl-1,2,4-triazin-3,5(2*H*,4*H*)-dione (**2**), (c) gHSQC spectrum.

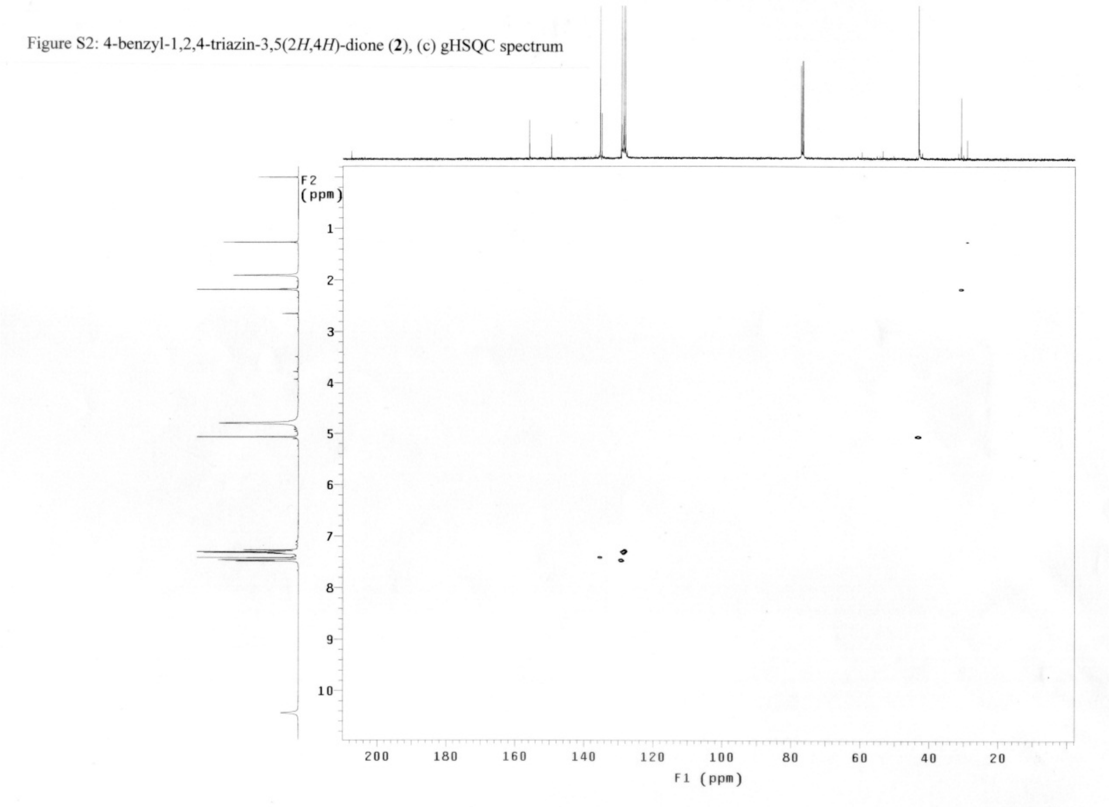

Figure S2: 4-Benzyl-1,2,4-triazin-3,5(2*H*,4*H*)-dione (**2**), (d) gHMBC spectrum.

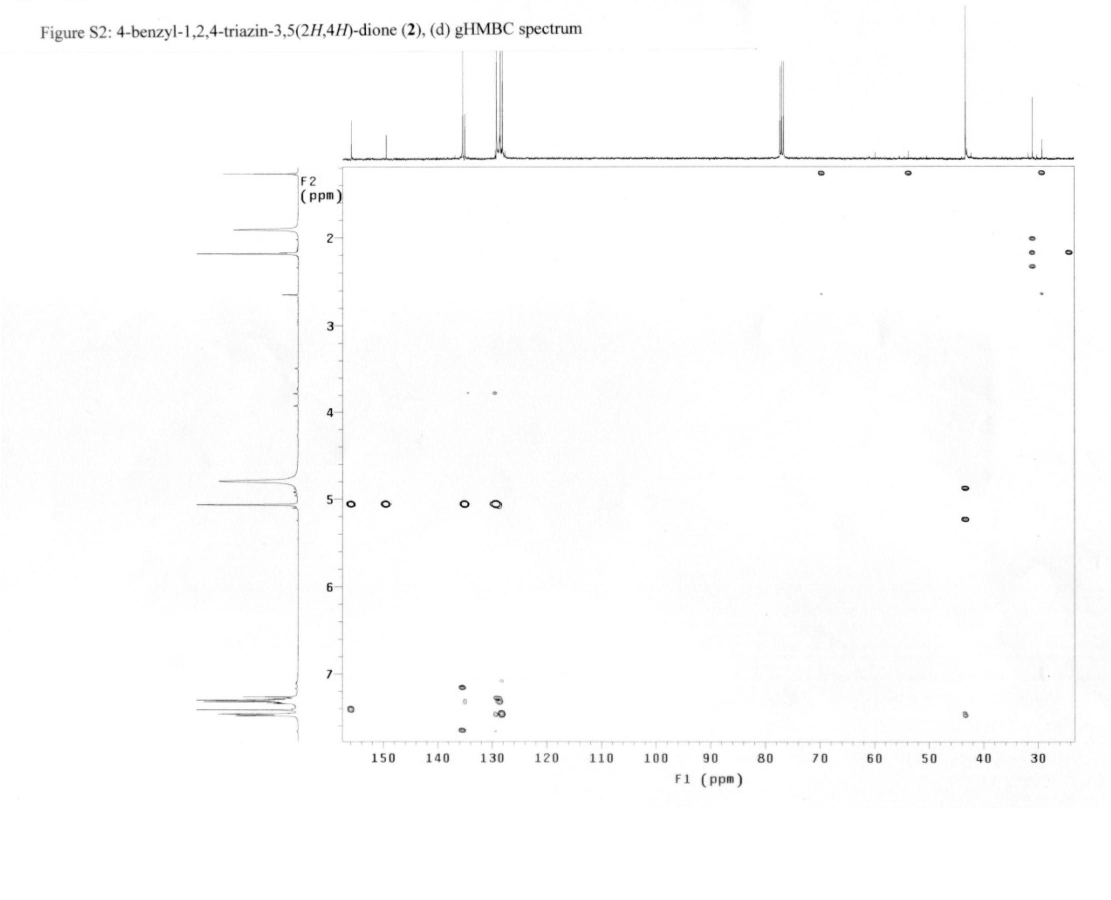

Figure S2: 4-Benzyl-1,2,4-triazin-3,5(2*H*,4*H*)-dione (**2**), (e) MS spectrum.

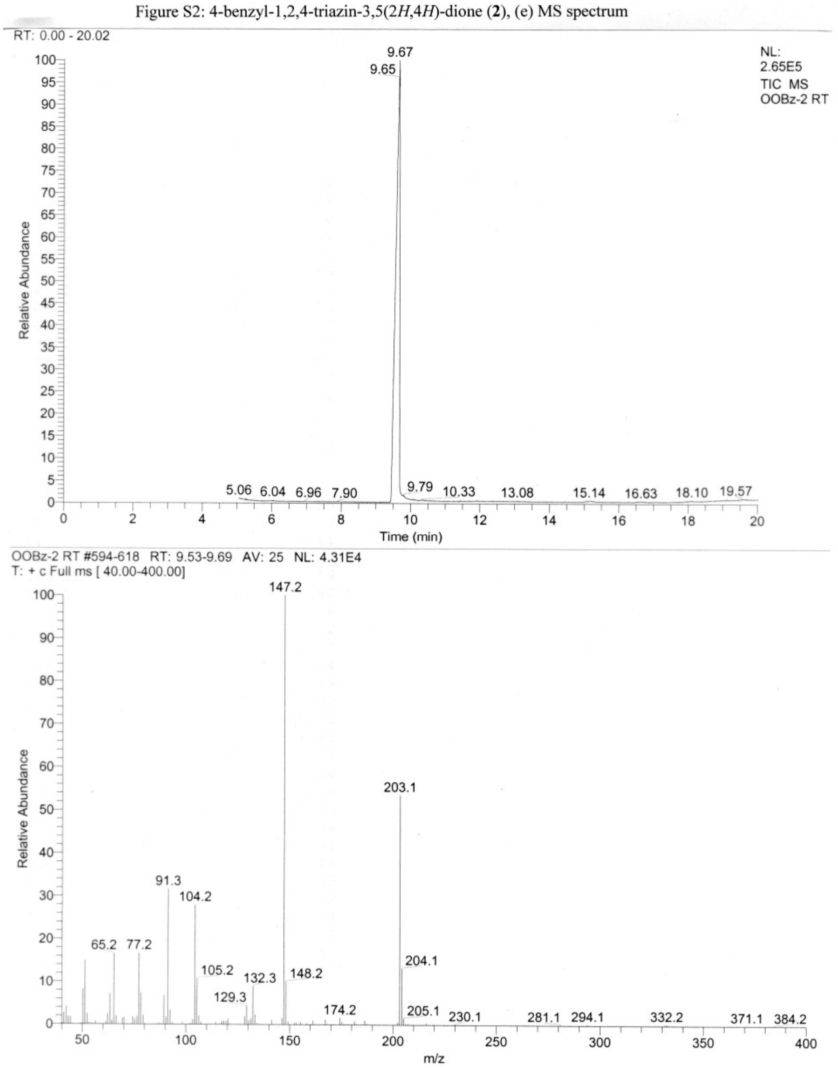

Figure S2: 4-Benzyl-1,2,4-triazin-3,5(2*H*,4*H*)-dione (**2**), (f) IR spectrum.

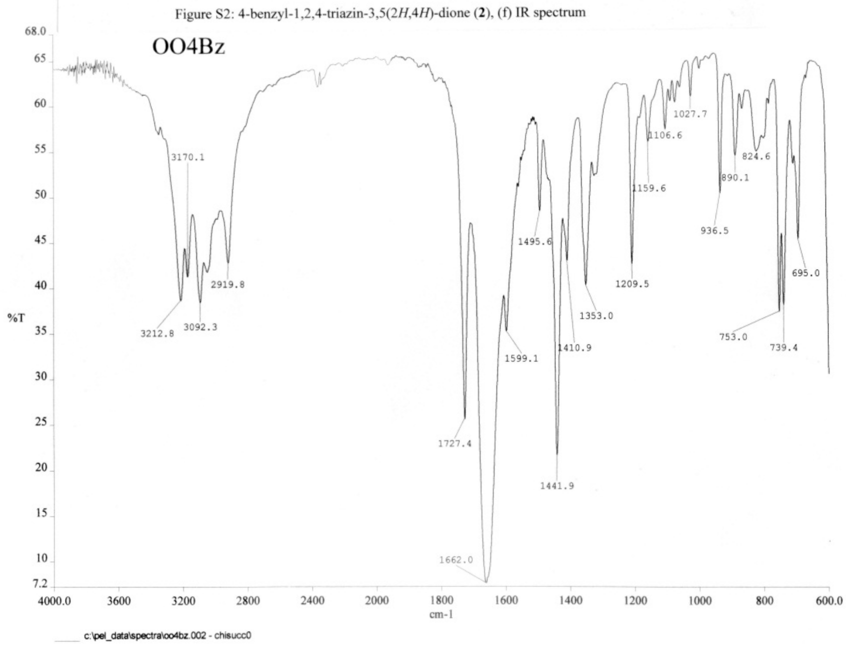

Figure S3: 2,4-Dibenzyl-1,2,4-triazin-3,5(2*H*,4*H*)-dione (**3**), (a) ^{1}H NMR spectrum.

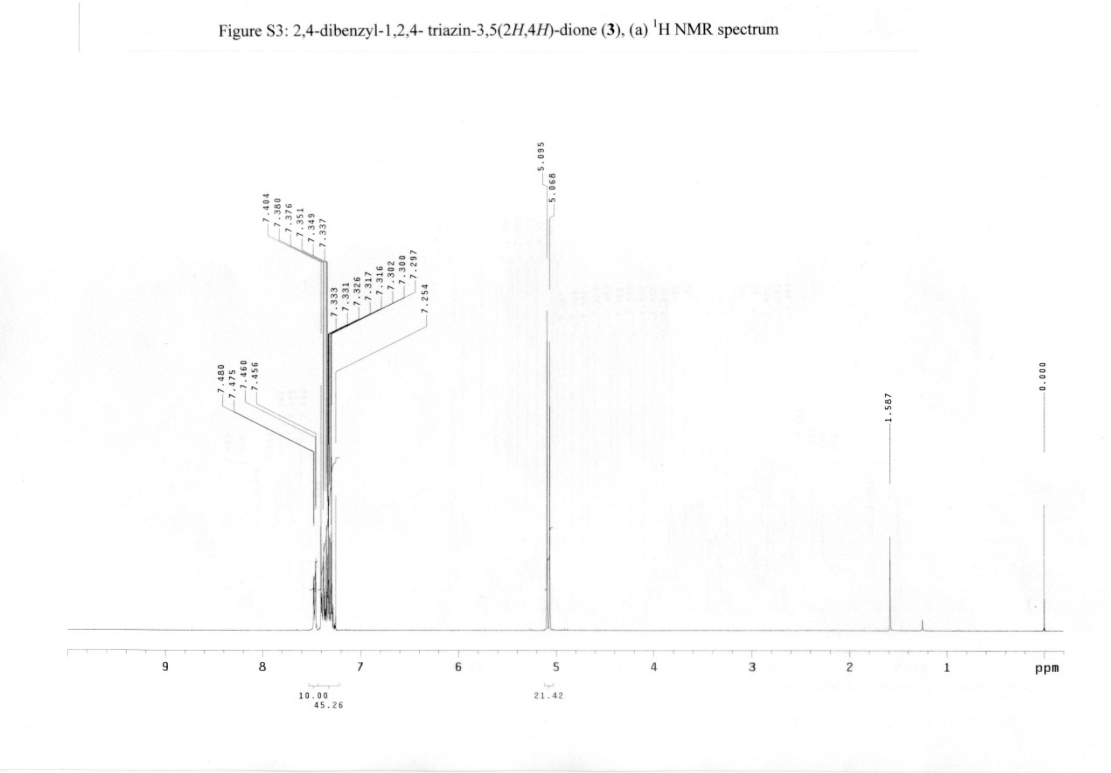

Figure S3: 2,4-Dibenzyl-1,2,4-triazin-3,5(2*H*,4*H*)-dione (**3**), (b) ^{13}C NMR spectrum.

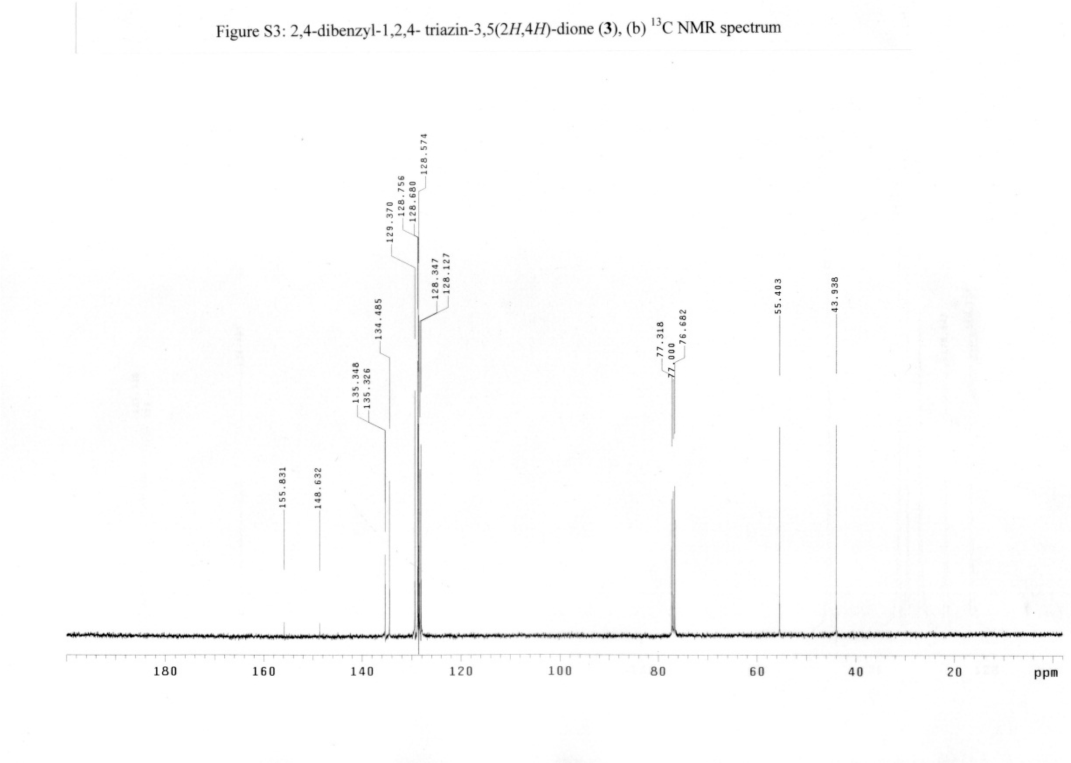

Figure S3: 2,4-Dibenzyl-1,2,4-triazin-3,5(2*H*,4*H*)-dione (**3**), (c) MS spectrum.

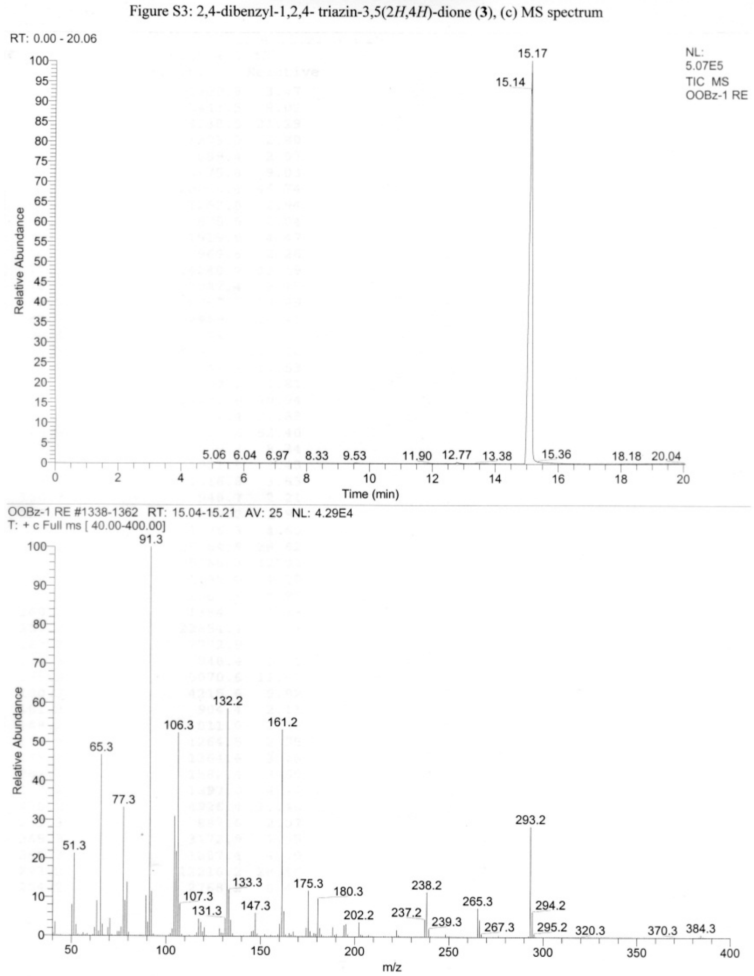

Figure S3: 2,4-Dibenzyl-1,2,4-triazin-3,5(2*H*,4*H*)-dione (**3**), (d) IR spectrum.

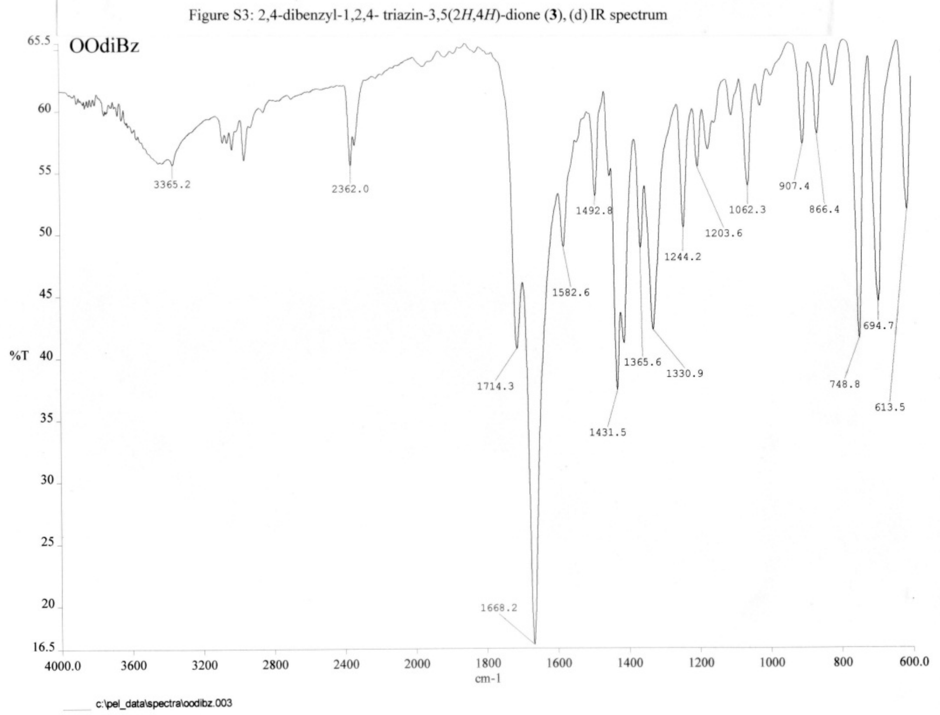

Supplement: Supplementary file 1 [file molecules-22-01924-s001.pdf]
